# Supplementary material for: Neurobehavioral Impacts of the Autism Risk Gene, WAC: Studies Involving C. elegans and Mice
Source: Mol Neurobiol. 2026 Jun 5;63(1):678. doi: 10.1007/s12035-026-05964-z (PMC13241405; doi:10.1007/s12035-026-05964-z)
Supplement: Supplementary file 1 — (DOCX 1.03 MB) [file 12035_2026_5964_MOESM1_ESM.docx]

**Supplementary Data**

***Table S1: Sequence of the primers used***

|  | **Gene Name** | **Primer Sequence 5’ to 3’** |
| --- | --- | --- |
| **1** | ***gpd-1* F** | AAAGTCATTCCGGAGCTGAAC GGA |
|  | ***gpd-1* R** | AGCGGCCTTGACTACCTTCTTGAT |
| **2** | ***cha-1* F** | GGGAAAGGGAAGAAACGA |
|  | ***cha-1* R** | GGACCACTGCACCATAC |
| **3** | ***unc-17* F** | GGCTCCCACCATTCTTTC |
|  | ***unc-17* R** | GCATCACCGATGTGGTATAG |
| **4** | ***ace-1 F*** | CAGAGTGAGGACACTTACTTTGGA |
|  | ***ace-1 R*** | CCCAAACCATTACAGCCAATTTCT |
| **5** | ***ace-2* F** | GCCCATTCGGATTTCTCTAC |
|  | ***ace-2* R** | AGCTGATTCTCCGAACAAAG |
| **6** | ***cho-1* F** | TCGATTCCACCGGATAAGA |
|  | ***cho-1* R** | CAAACATTGATGCTGCTGATAG |
| **7** | ***acr-2* F** | CAGGAATATGGGACGTGATTG |
|  | ***acr-2* R** | GAGAACCGTTGGGATGATAAG |
| **8** | ***acr-3* F** | TAATAGATGCACCGGGTTTG |
|  | ***acr-3* R** | ACTGGATTCTGCTGGTAAATA |
| **9** | ***acr-12* F** | ACGGGTAGATATGTGGATTTG |
|  | ***acr-12* R** | CGTTCGGATGTCAAGGATAG |
| **10** | ***lev-1* F** | CGCAGAGACGAAGAGATTAC |
|  | ***lev-1* R** | CTGATGGAGCAGTGAAGATTAT |
| **11** | ***lev-8* F** | GTGGATACCACAACGGATAAG |
|  | ***lev-8* R** | GAACTGGTCTGACAGCTTTAT |
| **12** | ***lev-10* F** | ACGACACATCCAGCAAAG |
|  | ***lev-10* R** | GTCTCTCGATTGCTCTCAAC |
| **13** | ***unc-29* F** | CGAGGACCAAGAACTCATC |
|  | ***unc-29* R** | ACTGTCCAACTCCTGGTA |
| **14** | ***unc-38* F** | CGCTGACAGCAACTACA |
|  | ***unc-38* R** | CAGGAGCCGAACTTCAA |
| **15** | ***unc-50* F** | CATCCCAGTCACCGATTT |
|  | ***unc-50* R** | TGAGAGCTTGGCGAATG |
| **16** | ***unc-63* F** | GGTCATCATAGCAAACCAAATC |
|  | ***unc-63* R** | GAGTTGTCGCGTGGTAAA |
| **17** | ***Wac F*** | CAGATGATTGGTCTGAGCACATTAG |
|  | ***Wac R*** | TTTCAAGCCACTCTTTTGGTTTCT |

**Figure S1**


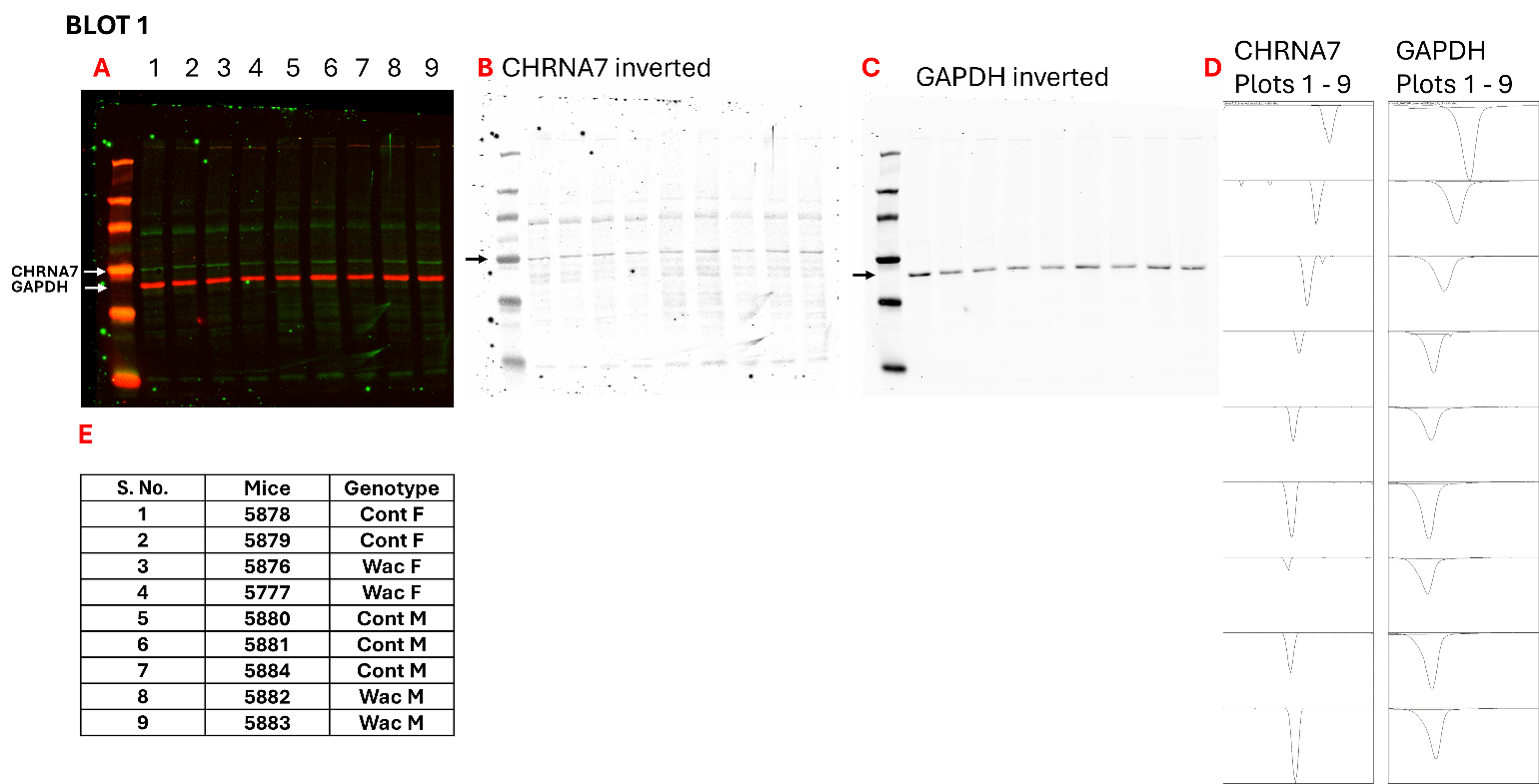


**Figure S2**


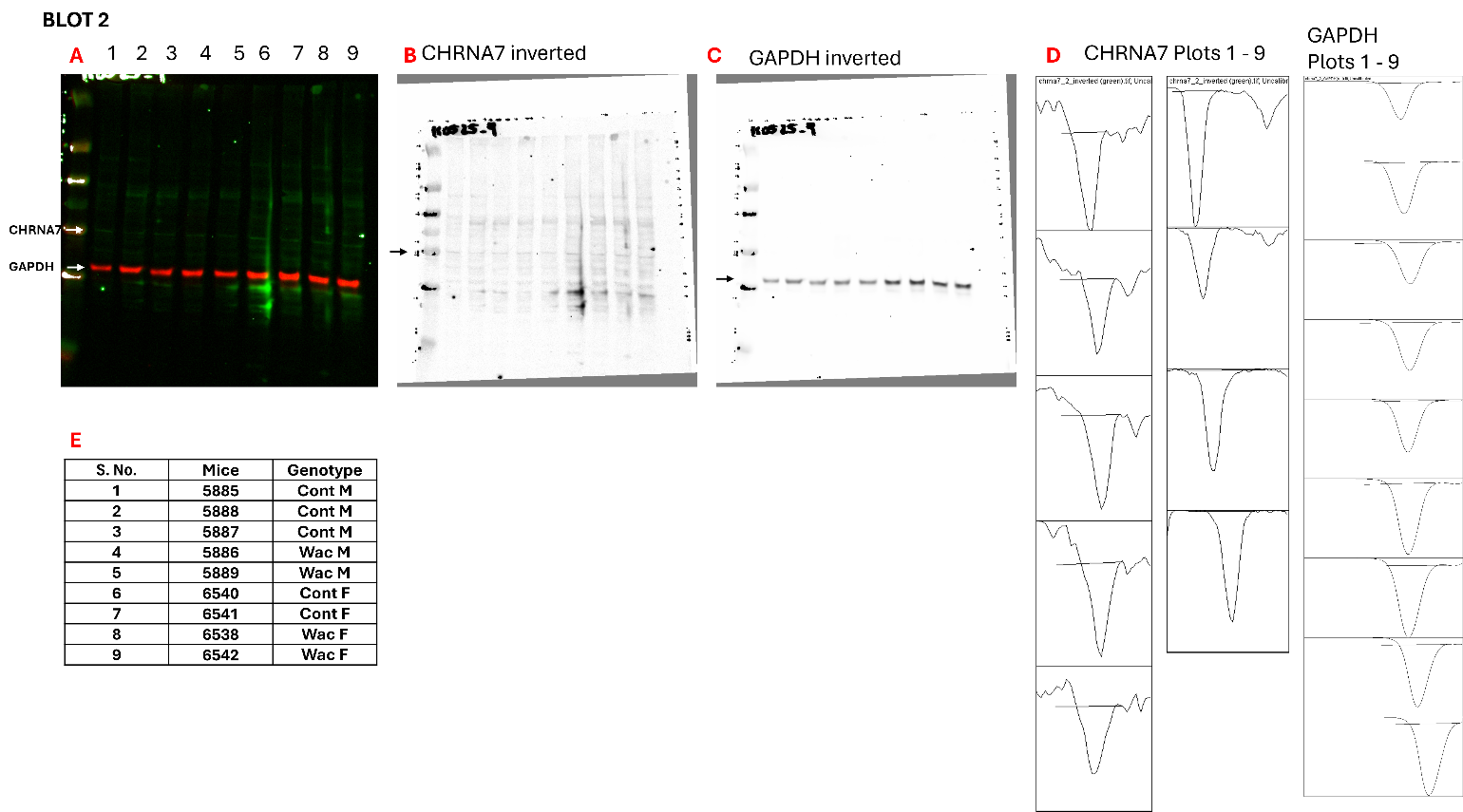


**Figure Legends**

**Figure S1: Blot 1:** Cortical tissues from Wac mutant and wild type mice were probed for CHRNA7 and GAPDH using respective antibodies. Raw immunoblot shown with protein bands indicated using white arrows (**A**). The blots were inverted using ImageJ, followed by densitometry for CHRNA7 (B) and GAPDH (**C**). Respective plots generated for CHRNA7 and GAPDH (**D**). Animal coding, sex, and genotype are shown in **E**.

**Figure S2: Blot 2:** Cortical tissues from Wac mutant and wild type mice were probed for CHRNA7 and GAPDH using respective antibodies. Raw immunoblot shown with protein bands indicated using white arrows (**A**). The blots were inverted using ImageJ, followed by densitometry for CHRNA7 (B) and GAPDH (**C**). Respective plots generated for CHRNA7 and GAPDH (**D**). Animal coding, sex, and genotype are shown in **E**.

**References**

1. Duerr JS, McManus JR, Crowell JA, Rand JB. Analysis of Caenorhabditis elegans acetylcholine synthesis mutants reveals a temperature-sensitive requirement for cholinergic neuromuscular function. Genetics. 2021;218(4). doi: 10.1093/genetics/iyab078. PubMed PMID: 34028515; PMCID: PMC9335933.

2. Rand JB. Genetic analysis of the cha-1-unc-17 gene complex in Caenorhabditis. Genetics. 1989;122(1):73-80. doi: 10.1093/genetics/122.1.73. PubMed PMID: 2731735; PMCID: PMC1203695.

3. Johnson CD, Rand JB, Herman RK, Stern BD, Russell RL. The acetylcholinesterase genes of C. elegans: Identification of a third gene (<em>ace-3</em>) and mosaic mapping of a synthetic lethal phenotype. Neuron. 1988;1(2):165-73. doi: 10.1016/0896-6273(88)90201-2.

4. Matthies DS, Fleming PA, Wilkes DM, Blakely RD. The Caenorhabditis elegans choline transporter CHO-1 sustains acetylcholine synthesis and motor function in an activity-dependent manner. J Neurosci. 2006;26(23):6200-12. doi: 10.1523/JNEUROSCI.5036-05.2006. PubMed PMID: 16763028; PMCID: PMC6675188.

5. Barbagallo B, Prescott HA, Boyle P, Climer J, Francis MM. A dominant mutation in a neuronal acetylcholine receptor subunit leads to motor neuron degeneration in Caenorhabditis elegans. J Neurosci. 2010;30(42):13932-42. doi: 10.1523/JNEUROSCI.1515-10.2010. PubMed PMID: 20962215; PMCID: PMC2965043.

6. Stawicki TM, Takayanagi-Kiya S, Zhou K, Jin Y. Neuropeptides function in a homeostatic manner to modulate excitation-inhibition imbalance in C. elegans. PLoS Genet. 2013;9(5):e1003472. Epub 20130502. doi: 10.1371/journal.pgen.1003472. PubMed PMID: 23658528; PMCID: PMC3642046.

7. Baylis HA, Matsuda K, Squire MD, Fleming JT, Harvey RJ, Darlison MG, Barnard EA, Sattelle DB. ACR-3, a Caenorhabditis elegans nicotinic acetylcholine receptor subunit. Molecular cloning and functional expression. Recept Channels. 1997;5(3-4):149-58. PubMed PMID: 9606719.

8. Petrash HA, Philbrook A, Haburcak M, Barbagallo B, Francis MM. ACR-12 ionotropic acetylcholine receptor complexes regulate inhibitory motor neuron activity in Caenorhabditis elegans. J Neurosci. 2013;33(13):5524-32. doi: 10.1523/JNEUROSCI.4384-12.2013. PubMed PMID: 23536067; PMCID: PMC3645261.

9. Philbrook A, Barbagallo B, Francis MM. A tale of two receptors: Dual roles for ionotropic acetylcholine receptors in regulating motor neuron excitation and inhibition. Worm. 2013;2(3):e25765. Epub 20130717. doi: 10.4161/worm.25765. PubMed PMID: 24778941; PMCID: PMC3875653.

10. Culetto E, Combes D, Fedon Y, Roig A, Toutant JP, Arpagaus M. Structure and promoter activity of the 5' flanking region of ace-1, the gene encoding acetylcholinesterase of class A in Caenorhabditis elegans. J Mol Biol. 1999;290(5):951-66. doi: 10.1006/jmbi.1999.2937. PubMed PMID: 10438595.

11. Fleming JT, Squire MD, Barnes TM, Tornoe C, Matsuda K, Ahnn J, Fire A, Sulston JE, Barnard EA, Sattelle DB, Lewis JA. Caenorhabditis elegans levamisole resistance genes lev-1, unc-29, and unc-38 encode functional nicotinic acetylcholine receptor subunits. J Neurosci. 1997;17(15):5843-57. doi: 10.1523/JNEUROSCI.17-15-05843.1997. PubMed PMID: 9221782; PMCID: PMC6573193.

12. Towers PR, Edwards B, Richmond JE, Sattelle DB. The Caenorhabditis elegans lev-8 gene encodes a novel type of nicotinic acetylcholine receptor alpha subunit. J Neurochem. 2005;93(1):1-9. doi: 10.1111/j.1471-4159.2004.02951.x. PubMed PMID: 15773900.

13. Davis AN, Tanis JE. Measuring Caenorhabditis elegans Sensitivity to the Acetylcholine Receptor Agonist Levamisole. J Vis Exp. 2022(184). Epub 20220607. doi: 10.3791/64056. PubMed PMID: 35758705; PMCID: PMC10016203.

14. Culetto E, Baylis HA, Richmond JE, Jones AK, Fleming JT, Squire MD, Lewis JA, Sattelle DB. The Caenorhabditis elegans unc-63 gene encodes a levamisole-sensitive nicotinic acetylcholine receptor alpha subunit. J Biol Chem. 2004;279(41):42476-83. Epub 20040727. doi: 10.1074/jbc.M404370200. PubMed PMID: 15280391.

15. Abiusi E, D'Alessandro M, Dieterich K, Quevarec L, Turczynski S, Valfort AC, Mezin P, Jouk PS, Gut M, Gut I, Bessereau JL, Melki J. Biallelic mutation of UNC50, encoding a protein involved in AChR trafficking, is responsible for arthrogryposis. Hum Mol Genet. 2017;26(20):3989-94. doi: 10.1093/hmg/ddx288. PubMed PMID: 29016857.
